# The Cellular Protein CAD is Recruited into Ebola Virus Inclusion Bodies by the Nucleoprotein NP to Facilitate Genome Replication and Transcription

**DOI:** 10.3390/cells9051126

**Published:** 2020-05-01

**Authors:** Janine Brandt, Lisa Wendt, Bianca S. Bodmer, Thomas C. Mettenleiter, Thomas Hoenen

**Affiliations:** Institute of Molecular Virology and Cell Biology, Friedrich-Loeffler-Institut, 17493 Greifswald-Insel Riems, Germany; janine.brandt@fli.de (J.B.); lisa.wendt@fli.de (L.W.); bianca.bodmer@fli.de (B.S.B.); ThomasC.Mettenleiter@fli.de (T.C.M.)

**Keywords:** Ebola virus, filovirus, inclusion bodies, CAD, pyrimidine synthesis

## Abstract

Ebola virus (EBOV) is a zoonotic pathogen causing severe hemorrhagic fevers in humans and non-human primates with high case fatality rates. In recent years, the number and extent of outbreaks has increased, highlighting the importance of better understanding the molecular aspects of EBOV infection and host cell interactions to control this virus more efficiently. Many viruses, including EBOV, have been shown to recruit host proteins for different viral processes. Based on a genome-wide siRNA screen, we recently identified the cellular host factor carbamoyl-phosphate synthetase 2, aspartate transcarbamylase, and dihydroorotase (CAD) as being involved in EBOV RNA synthesis. However, mechanistic details of how this host factor plays a role in the EBOV life cycle remain elusive. In this study, we analyzed the functional and molecular interactions between EBOV and CAD. To this end, we used siRNA knockdowns in combination with various reverse genetics-based life cycle modelling systems and additionally performed co-immunoprecipitation and co-immunofluorescence assays to investigate the influence of CAD on individual aspects of the EBOV life cycle and to characterize the interactions of CAD with viral proteins. Following this approach, we could demonstrate that CAD directly interacts with the EBOV nucleoprotein NP, and that NP is sufficient to recruit CAD into inclusion bodies dependent on the glutaminase (GLN) domain of CAD. Further, siRNA knockdown experiments indicated that CAD is important for both viral genome replication and transcription, while substrate rescue experiments showed that the function of CAD in pyrimidine synthesis is indeed required for those processes. Together, this suggests that NP recruits CAD into inclusion bodies via its GLN domain in order to provide pyrimidines for EBOV genome replication and transcription. These results define a novel mechanism by which EBOV hijacks host cell pathways in order to facilitate genome replication and transcription and provide a further basis for the development of host-directed broad-spectrum antivirals.

## 1. Introduction

Ebola virus (EBOV) is a zoonotic pathogen belonging to the genus *Ebolavirus* within the order *Filoviridae*, and is the causative agent of severe hemorrhagic fevers in humans and non-human primates with high case fatality rates [1,2]. Increasing numbers of EBOV outbreaks in Africa highlight the importance of understanding the molecular mechanisms of the EBOV life cycle and virus-host cell interactions better in order to develop new countermeasures against this virus. EBOV possesses a non-segmented single-stranded RNA genome of negative polarity that forms a helical nucleocapsid in the center of virions together with the ribonucleoprotein (RNP) complex proteins. During assembly of the nucleocapsid, the RNA genome is tightly coated with the viral nucleoprotein (NP), which protects it from degradation and recognition by the cellular immune response [3]. During EBOV infection, NP-associated RNA genomes serve as templates for mRNA transcription and genome replication [4]. For viral replication, NP interacts with the polymerase cofactor VP35, which acts as a linker between NP and the RNA-dependent RNA polymerase L [5]. NP, VP35, and L are sufficient to facilitate EBOV genome replication, while for viral transcription the transcriptional activator VP30 is additionally required [6,7]. EBOV replication and transcription takes place in cytoplasmic inclusion bodies, which represent a characteristic feature of EBOV infections in cells [8,9]. Their formation can be driven by the expression of NP alone [5,10,11]. Due to the limited number of viral genes, successful genome replication and transcription is highly dependent on host cell factors, which play an important role during the EBOV life cycle. For instance, the host factor STAU1 has been shown to interact with multiple EBOV RNP components, and to redistribute into NP-induced or virus-induced inclusion bodies, suggesting that STAU1 plays a crucial role during viral RNA synthesis by facilitating the interaction between the viral genome and RNP proteins [12]. EBOV has also been shown to recruit SMYD3 into inclusion bodies, which modulates NP-VP30 interaction and enhances mRNA transcription [13]. Similarly, RBBP6 was found to influence EBOV replication by disrupting the interaction between NP and VP30 [14]. Importin-α7 was described as being required for the efficient formation of inclusion bodies [15]. Furthermore, several cellular kinases and phosphatases are known to localize in inclusion bodies to support EBOV replication and transcription [16,17,18]. Finally, we previously showed that EBOV NP recruits the nuclear RNA export factor 1 (NXF1) into inclusion bodies to facilitate viral mRNA export from these structures into the cytoplasm [19]. Despite this recent progress in our understanding of the interplay between host factors and EBOV, there remains a considerable need to identify and, more importantly, characterize further host factors required for EBOV replication to identify novel targets for antiviral drug development.

We previously performed a genome-wide siRNA screen using a minigenome system to identify potential host-directed targets [20]. In this system, a minigenome, i.e., a truncated version of the EBOV genome lacking all viral open reading frames (ORF) and consisting of a reporter gene (e.g., a luciferase or green fluorescent protein) flanked by the viral non-coding terminal leader and trailer regions, is expressed from a plasmid in mammalian cells together with the plasmids encoding the viral RNP proteins [6]. For initial transcription of the minigenome RNAs from the minigenome-encoding plasmids most existing EBOV minigenome systems use a T7 RNA polymerase (T7) promoter, and therefore require expression of T7 polymerase, which is usually provided via a T7-expressing plasmid that is cotransfected with the plasmids encoding the RNP proteins [6,21]. However, recently, an EBOV minigenome system using the cellular RNA polymerase II (Pol-II) for initial minigenome RNA transcription has also been established and shown to be more efficient at least in some cell types [22]. After initial transcription and encapsidation by RNP proteins, minigenome RNAs are recognized as authentic templates by the viral polymerase due to their leader and trailer regions, and are replicated and transcribed into mRNAs, which results in expression of the reporter protein. Thus, minigenome assays allow us to study viral genome replication and transcription, as well as viral protein expression, outside of maximum containment laboratories, simplifying the identification of host factors involved in these processes. By using this system, we recently identified the trifunctional protein carbamoyl-phosphate synthetase 2, aspartate transcarbamylase, and dihydroorotase (CAD) as being important for the EBOV life cycle [20].

CAD is an important component of the pyrimidine pathway that catalyzes the first three steps during the de novo biosynthesis of pyrimidine nucleotides using its four distinct enzymatic domains [23,24,25]. The first domain, glutaminase (GLN), initiates the pathway by catalyzing the hydrolysis of glutamine. This is followed by the synthesis of carbamoyl phosphate facilitated by the carbamoyl-phosphate synthetase (CPS). Carbamoyl phosphate is in turn the substrate for the aspartate transcarbamylase (ATC), which catalyzes the reaction of aspartate with carbamoyl phosphate to carbamoyl aspartate [26,27]. Finally, carbamoyl aspartate is converted to dihydroorotate by dihydroorotase (DHO) [28]. In response to cell growth and proliferation, CAD activity is upregulated by phosphorylation through MAP kinases at position Thr-456, while in resting cells Thr-456 is dephosphorylated [29]. Furthermore, CAD is known to primarily localize in the cytoplasm of resting cells, but in response to cell growth and Thr-456 phosphorylation a small fraction is translocated into nuclear compartments, suggesting a cellular function of CAD in the nucleus [30,31]. However, little is known about the role of CAD during virus infection, and particularly the role of CAD in the EBOV life cycle still needed to be further analyzed. Therefore, we wanted to characterize the interaction of CAD with EBOV on both a biochemical and functional level. Based on our results, we suggest that CAD is important for both genome replication and transcription due to its function in pyrimidine synthesis and that it is recruited into NP-induced and virus-induced inclusion bodies to facilitate the de novo biosynthesis of pyrimidine nucleotides.

## 2. Materials and Methods

### 2.1. Cell Lines

Human embryonic kidney cells (HEK 293T, Collection of Cell Lines in Veterinary Medicine CCLV-RIE 1018), African green monkey kidney cells (Vero E6, kindly provided by Stephan Becker, Philipps University Marburg), and human hepatocellular carcinoma cells (Huh7, kindly provided by Stephan Becker, Philipps University Marburg) were cultured in Dulbecco’s modified Eagle’s medium (DMEM; Thermo Fisher Scientific, Darmstadt, Germany) supplemented with 10% fetal calf serum (FCS), 100 U/mL penicillin, 100 µg/mL streptomycin (PS; Thermo Fisher Scientific), and 1× GlutaMAX (Thermo Fisher Scientific). All cells were incubated at 37°C and 5% CO_2_.

### 2.2. Plasmids and Cloning

Minigenome assay components, including expression plasmids coding for the EBOV RNP proteins, T7 polymerase, firefly luciferase, and a classical T7-driven monocistronic minigenome (pT7-1cis-EBOV-vRNA-nLuc) have been previously described [20,32]. A NanoLuc luciferase-expressing T7-driven replication-deficient minigenome was cloned from a classical minigenome expressing NanoLuc luciferase as a reporter by deletion of 55 nucleotides (nt) in the antigenomic replication promoter as previously described [32]. Based on this, a Pol-II-driven replication-deficient minigenome was generated by PCR to amplify a linear version of the replication-deficient minigenome flanked by hammerhead and hepatitis delta virus ribozymes using primers #4571 (5′-AGC TTA CGT GAC TAC TTC CTT CGG ATG CCC AGG TCG GAC CGC G-3′) and #4572 (5′-GAC CGG TAG AAA ACT GAT GAG TCC GTG AGG ACG AAA CGG AGT CTA GAC TCC GTC TTT TCC AGG AAT CCT TTT TGC AAC GTT TAT TCT G-3′). The linearized construct was subsequently inserted into pCAGGS. The CAD gene was cloned from 293T cells into pCAGGS, and deletion mutants and domains of CAD were then generated using PCR-based approaches. All constructs were first cloned into pCAGGS, followed by subcloning into a pCAGGS plasmid encoding an N-terminal FLAG/HA-tag (DYKDDDDKLDGGYPYDVPDYA) immediately upstream of a BsmBI cloning site, allowing a seamless insertion of the open reading frame of interest. The expression plasmid for N-terminally myc-tagged VP35 was constructed by cloning a myc-tag (EQKLISEEDL) immediately before the VP35 ORF. Detailed cloning strategies are available on request.

### 2.3. Antibodies

The anti-FLAG (clone M2) antibody used for immunofluorescence analyses (IFA), co-immuno precipitation (coIP), and Western blot analyses was purchased from Sigma-Aldrich (Munich, Germany) [F1804], and the anti-c-myc antibody used for IFA analysis was obtained from Thermo Fisher Scientific [A-21281]. Primary antibodies against NP (rabbit anti-EBOV NP polyclonal antibody), GAPDH (mouse anti-GAPDH clone 0411), and CAD (rabbit anti-CAD clone EP710Y) were ordered from IBT Bioservices (San Jose, USA; anti-NP [0301-012]), Santa Cruz (Heidelberg, Germany; anti-GAPDH [sc47724]), or Abcam (Cambridge, UK; anti-CAD [ab40800]). Secondary antibodies used for IFA analysis against mouse (Alexa Fluor 488 anti-mouse [A-11029]), rabbit (Alexa Fluor 568 anti-rabbit [A-11036]), and chicken IgY (Alexa Fluor 647 anti-chicken [A-21449]) were obtained from Thermo Fisher Scientific. For Western blotting, secondary antibodies against mouse (IRDye 680RD anti-mouse [926-68070]) and rabbit IgG (IRDye 800CW anti-rabbit [926-68071]) were purchased from Li-COR (Bad Homburg, Germany), while anti-mouse IgG (Kappa light chain) Alexa Fluor 680 [115-625-174] used for coIP analyses was ordered from Dianova (Hamburg, Germany).

### 2.4. Viruses

Zaire ebolavirus rec/COD/1976/Mayinga-rgEBOV (GenBank accession number KF827427.1), which is identical in sequence to the EBOV Mayinga isolate with the exception of four silent mutations as genetic markers [33], was used for all infection experiments. rgEBOV was propagated in VeroE6 cells and virus titers were determined by 50% tissue culture infectious dose (TCID50) assay. All work with the infectious virus was performed under BSL-4 conditions at the Friedrich-Loeffler-Institut (Federal Research Institute of Animal Health, Greifswald Insel-Riems, Germany) following approved standard operating procedures.

### 2.5. Chemical Compounds

100mM uridine or cytidine (both Sigma-Aldrich) stock solutions were prepared in dimethyl sulfoxide (DMSO) and further diluted in cell culture medium. Diluted pyrimidines or DMSO corresponding to 1% of the supernatant volume in 12-well plates was added to the cells at the time of transfection and after medium changes. All concentrations indicated in the figures are final concentrations.

### 2.6. siRNA Knockdown with EBOV Minigenomes and Pyrimidine Complementation

For siRNA knockdown of endogenous CAD, 293T cells were reverse transfected (i.e., transfected in suspension and subsequently seeded into plates) with 12 pmol pre-designed silencer select siRNAs (CAD-siRNA#1: s2320 [5′-GAG GGU CUC UUC UUA AGU A-3′]; CAD-siRNA#2: 117891 [5′-GCU AGC UGA GAA AAA CUU U-3′]; Negative Control siRNA #2; all Thermo Fisher Scientific) or a self-designed EBOV-anti-L siRNA [5′-UUU AUA UAC AGC UUC GUA CUU-3′] ordered from Eurofins Genomics (Ebersberg, Germany). Transfection was performed in 12-well plates using Lipofectamine RNAiMax (Thermo Fisher Scientific) following the manufacturer’s instructions. 48 h post-siRNA transfection, the cells were transfected using Transit-LT1 (Mirus Bio LLC, Madison, USA) with all minigenome assay components, i.e., pCAGGS-based expression plasmids for NP (62.5 ng), VP35 (62.5 ng), VP30 (37.5 ng), L (500 ng), codon-optimized T7-polymerase (125 ng), firefly luciferase (as a control, 125 ng), and the T7-driven monocistronic minigenome (pT7-EBOV-1cis-vRNA-nLuc; 125 ng). For analyses of vRNA and mRNA levels the control firefly luciferase was replaced with GFP (200 ng), and for the replication-deficient minigenome assay a Pol-II-driven replication-deficient minigenome (pCAGGS-EBOV-1cis-vRNA-nLuc-RdM) was used. Transfections were performed using Transit LT1 as previously described [32]. All samples were harvested 48 h post-transfection for either determination of reporter activity or RNA isolation (see below). For measuring the luciferase activity, cells were lysed for 10 min in 1x Lysis Juice (PJK, Kleinblittersdorf, Germany) at room temperature and lysates were cleared of cell debris by centrifugation for 3 min at 10,000× g. Then, 40 µL of the cleared lysates were added to either 40 µL of Beetle Juice (PJK) or NanoGlo Luciferase Assay Reagent (Promega, Madison, USA) in opaque 96-well plates and luminescence was measured using a Glomax Multi (Promega) microplate reader. NanoLuc luciferase activities were normalized to firefly luciferase activities.

### 2.7. RNA Isolation and RT-qPCR

RNA isolation from minigenome cell lysates was performed following the manufacturer’s instructions using a NucleoSpin RNA kit (Machery-Nagel, Düren, Germany). After RNA purification, all samples were treated with DNase (TURBO DNA-free kit; Thermo Fisher Scientific) following the manufacturer’s instructions to avoid plasmid contamination. For cDNA generation, RNA samples were incubated with an oligo(dT)-primer for mRNA quantification or with a strand-specific primer (5′-AGT GTG AGC TTC TAA AGC AAC C-3′) for vRNA quantification using the RevertAid Reverse Transcriptase (Thermo Fisher Scientific) following the manufacturer’s instructions. The subsequent qPCR was performed using a PowerUp SYBR Green Master Mix (Thermo Fisher Scientific) with 1 µL of cDNA and primers targeting either the reporter gene (5′-TTC AGA ATC TCG GG GTG TCC-3′, 5′-CGT AAC CCC GTC GAT TAC CA-3′), or GFP as a control (5′-CTT GTA CAG CTC GTC CAT GC-3′, 5′-CGA CAA CCA CTA CCT GAG CAC-3′). Values for vRNA and mRNA levels were normalized to control GFP mRNA levels.

### 2.8. Immunofluorescence Analysis

Huh7 cells, which are more suitable for IFA than 293T cells, were seeded on coverslips in 12-well plates and transfected 24 h later with 500 ng pCAGGS-EBOV-NP and 500 ng pCAGGS-FLAG-HA-CAD (or CAD mutants) and, in selected experiments, additionally with 500 ng pCAGGS-myc-VP35 as indicated. For a mock control, cells were transfected with pCAGGS. Transfection was performed using polyethylenimine (Sigma-Aldrich) following the manufacturer’s instructions. 48 h post-transfection, cells were fixed using 4% paraformaldehyde (Roth, Karlsruhe, Germany) in DMEM for 20 min and then treated with 1 M glycine (in phosphate-buffered saline^++^ (PBS with 0.9M Ca^2+^ and 0.5M Mg^2+^)) for 10 min. Then, cells were permeabilized with 0.1% Triton X-100 in PBS for another 10 min and incubated with 10% fetal calf serum (FCS) in PBS for 45 min. Primary antibodies (rabbit anti-EBOV-NP 1:500; mouse anti-FLAG 1:2500; chicken anti-myc 1:1200) were diluted in PBS with 10% FCS and cells were incubated for 1 h at room temperature with the prepared antibody solutions. Secondary antibodies (Alexa Fluor 488 anti-mouse 1:1200; Alexa Fluor 568 anti-rabbit 1:500; Alexa Fluor 647 anti-chicken 1:1200) were prepared as described for the primary antibodies. After 45 min of staining, cells were washed with PBS and water before mounting with ProLong Diamond Antifade mountant with 4′,6-diamidino-2-phenylindole (DAPI) (Thermo Fisher Scientific). Slides were analyzed by confocal laser scanning microscopy using a Leica SP5.

### 2.9. Infection of Transfected Huh7 Cells

To investigate the localization of CAD during EBOV infection, Huh7 cells were seeded in 8-well chambered slides (ibidi, Martinsried, Germany) and transfected as described above (immunofluorescence analysis) with 500 ng pCAGGS-FLAG/HA-CAD. At 48 h post-transfection, the transfected cells were infected with EBOV at an MOI of 1, and the samples were fixed 16 h post-infection in 10% formalin twice overnight prior to removal from the BSL4 facility and immunofluorescence analysis.

### 2.10. Co-Immunoprecipitation of Viral Proteins

CoIPs were performed as previously described [19]. Briefly, 293T cells were seeded in 6-well plates and transfected with expression plasmids encoding FLAG/HA-tagged CAD and EBOV-NP using Transit LT-1 (Mirus Bio LLC) following the manufacturer’s instructions. The medium was changed after 24 h and the cells were harvested 48 h post-transfection. For coIP, cells were lysed in 1 mL coIP lysis buffer (1% NP-40; 50 mM Tris pH 7.4; 167 mM NaCl in water) with protease inhibitors (cOmplete; Roche, Mannheim, Germany). To investigate a possible RNA dependency of the interaction between CAD and NP, 100 µg/mL RNase A (Machery-Nagel) were added to the samples. Subsequently, the samples were incubated rotating at 15 RPM for 2 h at 4 °C. Then, 150 µL of the cleared lysates were taken as an input control (representing a sixth of the complete pre-immune lysate and 20% of the sample used for immunoprecipitation) and subjected to acetone precipitation. The remaining 750 µL of cell lysate were mixed with the prepared bead-antibody solution (Dynabeads Protein G, Thermo Fisher Scientific; 1 µL anti-FLAG M2 antibody per 10 µL beads). Immunoprecipitation was performed for 10 min, as recommended by the manufacturer, at room temperature and rotation at 15 RPM. Then, samples were transferred to new tubes and boiled for 10 min at 99 °C. Input and coIP samples were analyzed by SDS-PAGE and Western blotting.

### 2.11. Western Blotting

For validation of CAD knockdown efficiency and analyses of coIP input and lysates, samples were subjected to SDS-PAGE and Western blotting as previously described [34]. FLAG-tagged CAD was detected using a monoclonal anti-FLAG antibody (1:2000), while NP, wild type CAD, and GAPDH were detected using anti-NP (1:1000), anti-CAD (1:250), and anti-GAPDH (1:1000) antibodies. As secondary antibodies, 680RD-coupled goat-anti-mouse, goat-anti-mouse-Alexa Fluor 680, and 800CW-coupled goat-anti-rabbit antibodies (1:14000) were used. Fluorescent signals were detected and quantified using an Odyssey CLx infrared imaging system (Li-Cor Biosciences). For knockdown quantification, CAD signals were normalized to GAPDH signals.

### 2.12. Statistical Analyses

One-way ANOVA with a Dunnett’s multiple comparisons test was performed using the GraphPad Prism 8.1.0 software.

## 3. Results

### 3.1. CAD Knockdown Affects Both EBOV Genome Replication and Transcription

**Figure 1:**
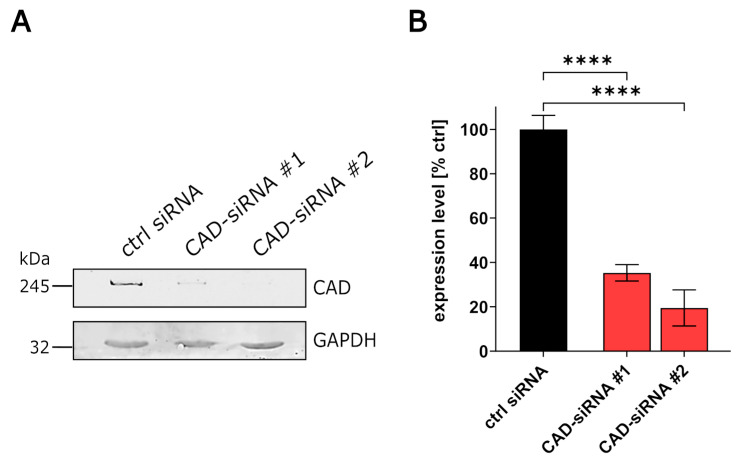
Quantification of CAD knockdown. (**A**) Analysis of CAD knockdown. 293T cells were transfected with siRNAs targeting CAD (CAD-siRNA), or a negative control (ctrl siRNA). The cells were harvested 48 h post-transfection and the lysates were subjected to SDS-PAGE and Western blotting. (**B**) Quantification of CAD knockdown. The Western blot signals for CAD knockdown (as shown in Figure 1A) were measured and normalized to the GAPDH signals. The negative control (ctrl siRNA) was set to 100% and the efficiency of CAD knockdown was calculated (**** *p* ≤ 0.0001).

Using a genome-wide siRNA screen, we previously identified CAD to be important for EBOV RNA synthesis and/or viral protein expression [20]. However, since only the effect of CAD knockdown on the sum of these processes had been tested, we now analyzed the role of CAD on individual aspects of the EBOV life cycle. As a first step, we assessed the efficiency of endogenous CAD knockdown using two different siRNAs via quantitative Western blotting, which revealed a 60% to 80% reduction in endogenous CAD expression levels for the two siRNAs (Figure 1A,B).

**Figure 2:**
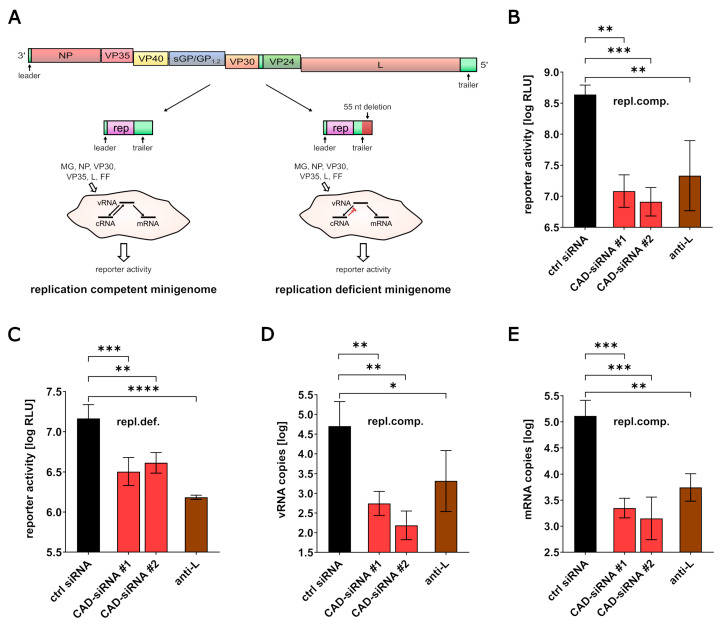
Influence of CAD knockdown on the Ebola virus life cycle. (**A**) Replication-competent and -deficient minigenome systems. The full-length genome structure of EBOV, as well as replication-competent and -deficient minigenomes derived from this full-length genome, are shown. Abbrevations: MG: minigenome, rep: reporter; FF: Firefly luciferase. Figure modified from [35] under CC BY 4.0 license. (**B**) Influence of CAD knockdown on EBOV RNA synthesis. 293T cells were transfected with siRNAs targeting either CAD (CAD-siRNA), EBOV-L (anti-L), or a negative control (ctrl siRNA). 48 h post-transfection, cells were transfected with all the components required for a replication-competent minigenome assay (repl.comp.). Another 48 h later, cells were harvested and the reporter activity was measured. (**C**) Analysis of CAD knockdown on EBOV transcription and gene expression. 293T cells were transfected with siRNAs targeting either CAD (CAD-siRNA), EBOV-L (anti-L), or a negative control (ctrl siRNA). 48 h post-transfection, cells were transfected with all the components required for a replication-deficient minigenome assay (repl.def.). Another 48 h later, cells were harvested and the reporter activity was measured. (**D**) Impact of CAD knockdown on EBOV replication. Cells were treated as described in 2B. After cell harvesting, RNA was extracted from the cell lysates and RT-qPCR for vRNA was performed. (**E**) Influence of CAD knockdown on EBOV mRNA levels. Cells were treated as described in 2B. After cell harvesting, RNA was extracted from cell lysates and RT-qPCR for mRNA was performed. The means and standard deviations of 3 independent experiments are shown for each panel. Asterisks indicate *p*-values from a one-way ANOVA (* *p* ≤ 0.05; ** *p* ≤ 0.01; *** *p* ≤ 0.001; **** *p* ≤ 0.0001; ns: *p* > 0.05).

Next, we performed a classical minigenome assay (Figure 2A) in connection with an siRNA knockdown of CAD. As previously shown, knockdown of CAD led to a 40 to 53-fold reduction in reporter activity, verifying an influence of CAD on EBOV viral RNA synthesis and protein expression (Figure 2B) [20]. In order to identify whether CAD knockdown affects transcription and/or protein expression independent of replication, we next used a replication-deficient minigenome system [32]. In contrast to a replication-competent minigenome, the replication-deficient minigenome lacks 55 nt in the antigenomic replication promoter leading to a block of minigenome vRNA replication, while minigenome transcription still takes place [32]. However, when using this system, which is based on T7-driven initial transcription of minigenomes, we observed a very low dynamic range between our controls, which made it difficult to evaluate a possible influence of CAD knockdown (Figure S1). Therefore, in order to increase the dynamic range of this system, we generated a Pol-II-driven replication-deficient minigenome that resulted in a ~10-fold higher dynamic range (Figure S1). Using this system, CAD knockdown resulted in a clear reduction in reporter activity, indicating that CAD is important for EBOV transcription and/or protein expression independent of viral genome replication (Figure 2C).

To further dissect the influences of CAD on viral genome replication, mRNA transcription, and later steps of viral protein expression, we performed classical minigenome assays in the context of an siRNA knockdown of CAD and measured vRNA and mRNA levels in cell lysates using RT-qPCR. For this, we used either an oligo-dT primer for reverse transcription of mRNAs, or a strand-specific primer for reverse transcription of vRNA, followed by qPCR against the reporter gene. CAD siRNA-treated cells showed a strong reduction in both vRNA and mRNA levels in comparison to the control cells, demonstrating that CAD is important for both EBOV transcription and viral genome replication (Figure 2D,E).

### 3.2. The Effect of CAD Knockdown Can Be Compensated for by Exogenous Pyrimidines

**Figure 3:**
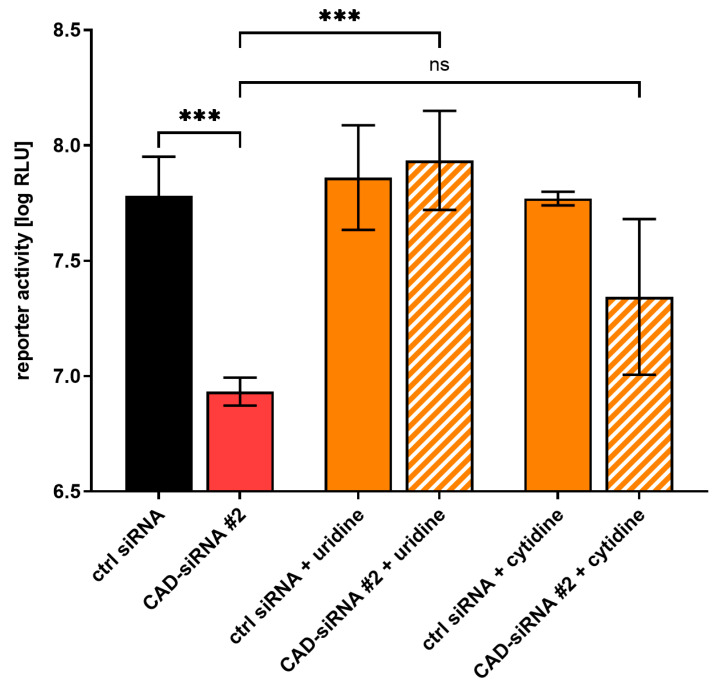
Supplementation of pyrimidines compensates for the effect of CAD knockdown. 293T cells were transfected with siRNAs targeting CAD (CAD-siRNA) or a negative control (ctrl siRNA). 48 h post-transfection, the cells were transfected with all the components required for a replication-competent minigenome assay and treated with 1 mM pyrimidines, either uridine or cytidine. Another 48 h later, the cells were harvested and the reporter activity was measured. The means and standard deviations of 3 independent experiments are shown. Asterisks indicate *p*-values from a one-way ANOVA (*** 0.0001 < *p* ≤ 0.001; ns: *p* > 0.05).

As CAD is an important component for pyrimidine synthesis [23], we wanted to investigate the effect of providing exogenous pyrimidines on EBOV transcription and replication during siRNA knockdown of CAD. To this end, we performed an siRNA-mediated knockdown of CAD with EBOV minigenomes and treated the cells with 1 mM of either uridine or cytidine. Complementation of uridine resulted in reporter activities similar to the positive controls, indicating that the effect of CAD knockdown on EBOV genome replication and transcription is due to a lack of pyrimidines (Figure 3). When providing cytidine, a similar rescue effect was seen, albeit less pronounced, possibly because cytidine is not metabolized into uridine, whereas exogenous uridine can be metabolized into cytidine during natural pyrimidine synthesis.

### 3.3. CAD Colocalizes with NP-Induced Inclusion Bodies

**Figure 4:**
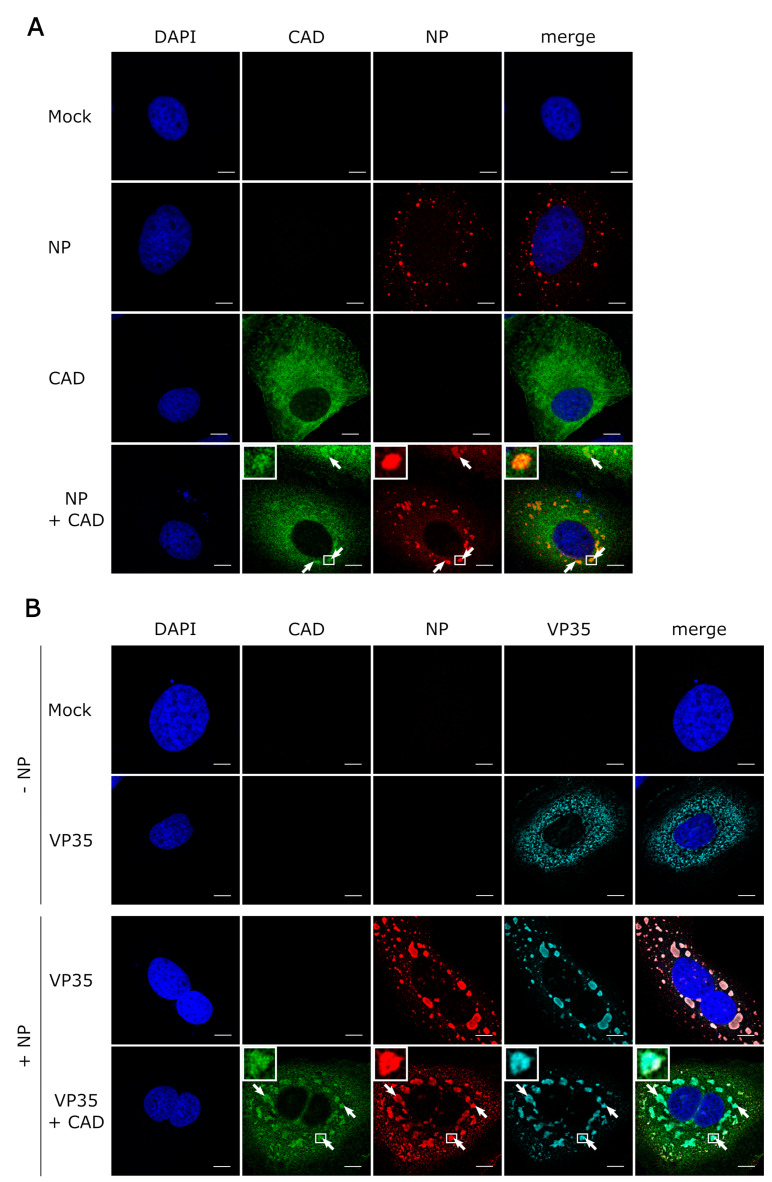
Recruitment of CAD into NP-induced inclusion bodies. (**A**) Colocalization between CAD and NP-induced inclusion bodies. Huh7 cells were transfected with plasmids encoding FLAG/HA-CAD and EBOV-NP as indicated. 48 h post-transfection, the cells were fixed with 4% paraformaldehyde and permeabilized with 0.1% Triton X-100. FLAG-tagged CAD (shown in green) was detected using an anti-FLAG antibody and NP (shown in red) was stained with anti-EBOV NP antibodies. (**B**) Recruitment of CAD into inclusion bodies occurs in the presence of VP35. Huh7 cells were transfected with plasmids encoding FLAG/HA-CAD, EBOV-NP, and myc-EBOV-VP35 as indicated. 48 h post-transfection, the cells were fixed with 4% PFA and permeabilized with 0.1% Triton X-100. FLAG-tagged CAD (shown in green) was detected using an anti-FLAG antibody, NP (shown in red) was stained with anti-EBOV NP antibodies, and myc-tagged VP35 (shown in turquoise) with an anti-myc antibody. The nuclei were stained with DAPI (shown in blue), and the cells were visualized by confocal laser scanning microscopy. The scale bars indicate 10 µm. The arrows point out colocalization, and the insets show magnifications of the indicated areas. Merge shows an overlay of all three channels.

**Figure 5:**
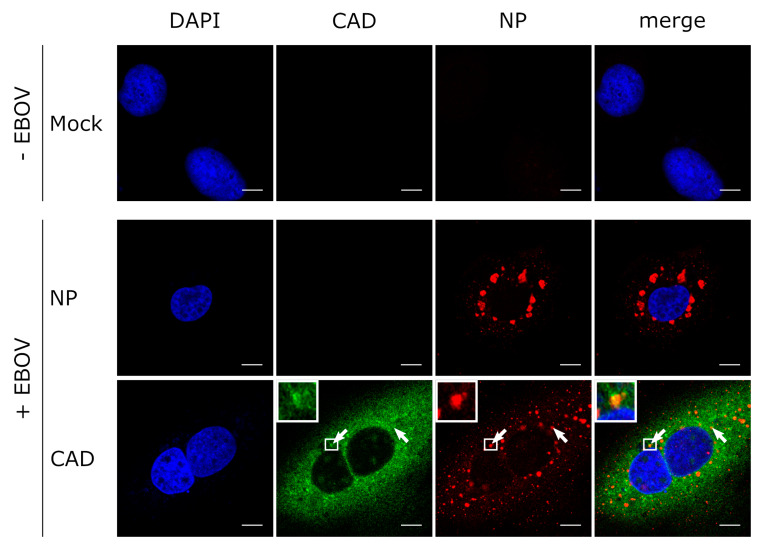
CAD localizes in EBOV inclusion bodies. Huh7 cells were transfected with a plasmid encoding FLAG/HA-CAD. 48 h post-transfection, the cells were infected with rgEBOV at an MOI of 1. After incubation for 16 h, the cells were fixed with 10% formalin and permeabilized with Triton X-100. CAD (shown in green) was detected with an anti-FLAG antibody and NP (shown in red) with an anti-NP antibody. The nuclei were stained with DAPI (shown in blue), and the cells were visualized by confocal laser scanning microscopy. Scale bars indicate 10 µm. The arrows point out colocalization, and the insets show magnifications of the indicated areas. Merge shows an overlay of all three channels.

Similar to other negative-sense RNA viruses, EBOV and in particular its nucleoprotein NP is known to induce the formation of cytoplasmic inclusion bodies, which are sites of viral genome replication and transcription [8,9]. Since we had shown that CAD is important for EBOV replication and transcription, we wanted to investigate whether the presence of inclusion bodies has an influence on the intracellular distribution of CAD, and in particular whether recruitment of CAD into NP-induced inclusion bodies can be detected. As previously reported, expression of only NP resulted in the formation of inclusion bodies, predominantly in the perinuclear region [5,10,11], while sole expression of CAD led to an even distribution throughout the cytoplasm, with small amounts of CAD present in the nucleus [30] (Figure 4A). During coexpression of NP and CAD we observed relocalization of CAD into NP-induced inclusion bodies (with clear accumulation in inclusion bodies in 70% of the cells, clear exclusion in 0%, and an unclear phenotype in 30%). When we additionally coexpressed VP35, which is involved in nucleocapsid formation during EBOV infection, together with NP [36], we observed a similar relocalization (Figure 4B). To confirm these results, we also performed experiments with infectious EBOV and stained the samples for NP as an inclusion body marker and CAD (Figure 5). Colocalization of CAD and inclusion bodies was still detectable, albeit not as apparent as under conditions of recombinant overexpression of NP and VP35. Taken together, these results suggest that CAD is recruited into viral inclusion bodies to provide sufficient amounts of pyrimidines for EBOV genome replication and transcription.

### 3.4. The GLN Domain of CAD Is Required for its Accumulation in Inclusion Bodies

**Figure 6:**
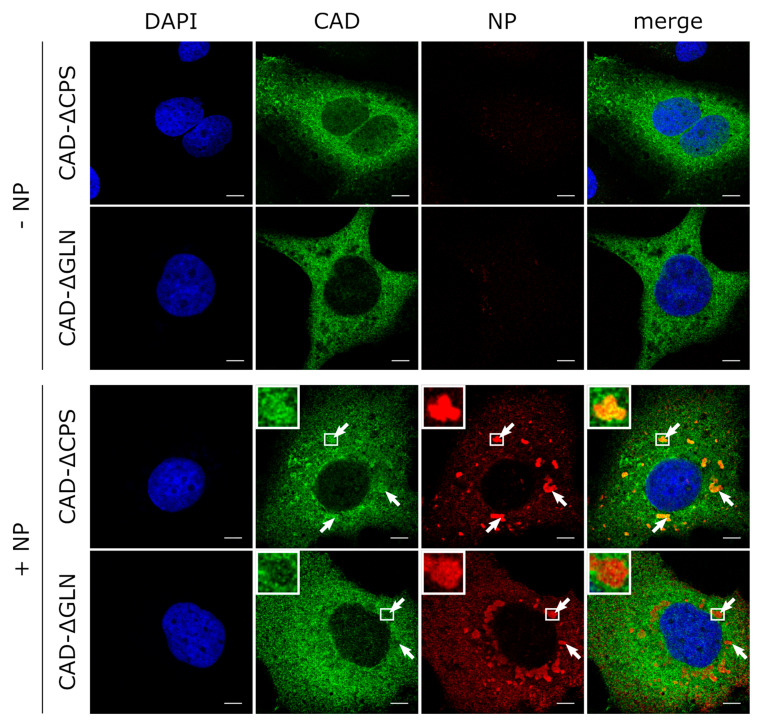
Recruitment of CAD deletion mutants into inclusion bodies. Huh7 cells overexpressing FLAG/HA-CAD-ΔGLN, FLAG/HA-CAD-ΔCPS and EBOV-NP, as indicated, were fixed with 4% PFA and permeabilized with 0.1% Triton X-100 48 h post-transfection. FLAG-tagged CAD (shown in green) was detected using an anti-FLAG antibody and NP (shown in red) was stained with EBOV anti-NP antibodies. The nuclei were stained with DAPI (shown in blue), and the cells were visualized by confocal laser scanning microscopy. Scale bars indicate 10 µm. The arrows point out inclusion bodies, and the insets show magnifications of the indicated areas. Merge shows an overlay of all three channels.

To assess the contribution of individual domains of CAD in its recruitment into NP-induced inclusion bodies, we focused on the GLN and the CPS domains. When we expressed deletion mutants lacking these domains, they showed a similar intracellular distribution compared to wild-type CAD when expressed alone in cells. During coexpression of NP and CAD-ΔCPS, we observed recruitment of this mutant into NP-driven inclusion bodies (with clear accumulation in inclusion bodies in 50% of the cells, clear exclusion in 0%, and an unclear phenotype in 50%), indicating that the CPS domain of CAD is not required for its accumulation in inclusion bodies (Figure 6). In stark contrast, when NP was expressed together with CAD-ΔGLN, colocalization with inclusion bodies was abolished (with clear accumulation in inclusion bodies in 0% of the cells, clear exclusion in 68%, and unclear phenotype in 32%), suggesting that the GLN domain is required for recruitment and accumulation in NP-induced inclusion bodies.

### 3.5. CAD Interacts with NP in an RNA-Independent Manner

**Figure 7:**
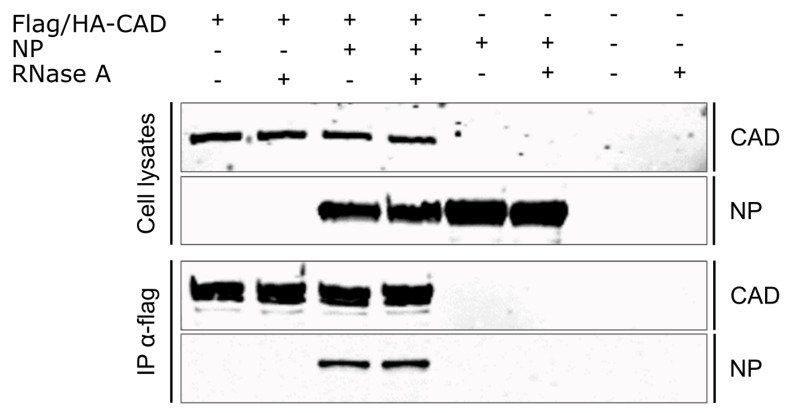
Interaction of CAD with NP. 293T cells were transfected with plasmids encoding FLAG/HA-CAD and EBOV-NP. 48 h post-transfection, the cells were lysed and treated with RNase A (100 µg/mL) or remained untreated. FLAG/HA-CAD was precipitated using anti-FLAG antibodies, and input and precipitates were analyzed via SDS-PAGE and Western blotting using anti-FLAG and anti-NP antibodies. In the CAD IP sample, several bands for CAD are visible, possibly due to posttranslational modifications that are not visible in the lysates because of the overall lower CAD signals in those samples.

As NP recruits CAD into EBOV inclusion bodies, we next assessed whether CAD interacts with NP. To this end, we performed coIP assays using FLAG-CAD expressed in the presence of NP by precipitating CAD with an anti-FLAG antibody and then detecting NP by Western blotting. We could readily co-precipitate NP with CAD, indicating that CAD is able to interact with NP (Figure 6). Because NP is an RNA-binding protein [37], we also tested whether this interaction between CAD and NP is RNA-dependent by treating the samples prior to coIP with RNase A. Under these conditions, we were still able to co-precipitate NP with CAD, demonstrating that the interaction between CAD and NP is not dependent on the presence of RNA (Figure 7).

## 4. Discussion

In this work, we identified CAD, an essential component of the de novo pyrimidine synthesis pathway, to be important for both EBOV genome replication and transcription, and demonstrated that the function of CAD in pyrimidine synthesis is responsible for this effect. Knockdown of CAD was also shown to affect replication and transcription of other viruses, e.g., hepatitis C viruses [38]. Furthermore, inhibitors of CAD, e.g., the antinucleoside N-phosphonacetyl-l-aspartate (PALA), which transiently inhibits the aspartate transcarbamylase activity of CAD, were effective in vitro against various viruses, including vaccinia virus and arenaviruses [39,40]. The fact that these compounds exhibit antiviral activity against a broad range of viruses qualifies CAD as a promising indirect antiviral target. However, whether PALA shows antiviral efficiency against EBOV remains to be investigated. Further, whether targeting viral RNA synthesis by inhibition of CAD will be synergistic with other inhibitors of EBOV RNA synthesis, such as remdesivir [41], will have to be addressed in future studies.

Our results are consistent with the fact that several pyrimidine synthesis inhibitors are effective against EBOV in vitro, underlining the importance of the pyrimidine pathway for these viruses [20,42]. Examples are the FDA-approved drug leflunomide and its active metabolite teriflunomide, as well as SW835, a racemic version of GSK983, which has been described to exhibit a broad-spectrum antiviral activity [20,42,43]. These compounds all impair de novo pyrimidine biosynthesis through inhibition of dihydroorotate dehydrogenase (DHODH), an enzyme downstream of CAD in the pyrimidine pathway. Interestingly, treatment with these inhibitors seems to have similar inhibitory effects on EBOV minigenome assays compared to the effect we observe for CAD knockdown, although CAD activity is not directly affected [20,42]. Provision of pyrimidines or upstream metabolites, e.g., orotic acid, reversed antiviral activity of all pyrimidine pathway inhibitors in EBOV minigenome assays, which is consistent with our observation that supplementation with pyrimidines restores reporter activity after CAD knockdown. Interestingly, inhibition of DHODH by using SW835 not only showed pyrimidine depletion, but also stimulated ISG (interferon-stimulated gene) expression, which contributes to the innate immune response [42]. However, currently, the mechanism behind this stimulation of the innate immune response by DHODH inhibitors remains incompletely understood and needs to be further analyzed. Further supporting the importance of CAD for the EBOV lifecycle is the fact that the de novo pyrimidine synthesis activity of CAD is a prerequisite for cell division, which has been suggested to be necessary for productive infection of cells with EBOV [44].

We were further able to show that CAD is recruited to EBOV inclusion bodies, which represent the site of EBOV replication and transcription [8,9]. Since we observed CAD recruitment into NP-induced inclusion bodies during expression of NP alone and detected an interaction of CAD with NP using CoIP studies, we suggest that this recruitment is mediated via an interaction of CAD with NP. So far, knowledge regarding direct interactions between CAD and the proteins of other viruses is limited, but Angeletti et al., showed that CAD recruits the preterminal protein (pTP) of adenoviruses to the site of adenovirus replication in the nuclear matrix via direct interaction. This interaction is believed to be required for anchorage of the adenovirus replication complex at the nuclear matrix in close proximity of the cellular factors required to segregate replicated and genomic viral DNA [45,46].

In the context of its cellular function, CAD has been shown to localize primarily in the cytoplasm, although small amounts can also be detected in the nucleus of dividing cells. Redistribution of CAD into nuclear compartments during cell growth and proliferation is believed to be in response to phosphorylation by MAP kinases at position Thr-456, which results in upregulation of the enzymatic activity of CAD [30]. Since NP is known to recruit a number of factors, including kinases and phosphatases, into inclusion bodies [16,17,18], it is possible that recruited CAD is activated in inclusion bodies in order to provide pyrimidines for EBOV replication and transcription. However, CAD lacking the CPS domain, which contains Thr-456, was still recruited into NP-induced inclusion bodies, excluding selective recruitment of Thr-456-phosphorylated and thus activated CAD into inclusion bodies.

Overall, we have shown that CAD is recruited into NP-induced and virus-induced inclusion bodies to provide sufficient amounts of pyrimidines for EBOV genome replication and transcription. Furthermore, we demonstrated that the GLN domain of CAD is required for recruitment into inclusion bodies. These findings increase our understanding of EBOV and its host cell interactions, and provide a basis for future identification of molecular targets for the development of novel therapies against this virus.

## Supplementary Materials

The following are available online at https://www.mdpi.com/2073-4409/9/5/1126/s1: Figure S1. Comparison of T7 and Pol-II-driven replication-deficient minigenomes.
