# Supplementary material for: The Cellular Protein CAD is Recruited into Ebola Virus Inclusion Bodies by the Nucleoprotein NP to Facilitate Genome Replication and Transcription
Source: Cells. 2020 May 1;9(5):1126. doi: 10.3390/cells9051126 (PMC7290923; doi:10.3390/cells9051126)
Supplement: Supplementary file 1 [file cells-09-01126-s001.pdf]

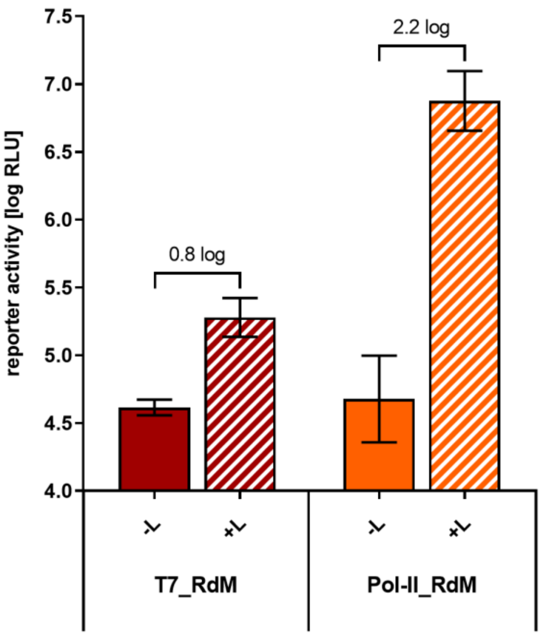

Figure S1. Comparison of T7 and Pol-II-driven replication-deficient minigenomes. 293T cells were transfected with plasmids encoding for all minigenome components (NP, VP35, VP30, polymerase L and/or T7) as well as either a T7-driven or Pol-II-driven minigenome. After 48 hours, cells were harvested and reporter activity was measured. As negative control, the plasmid encoding for the viral polymerase was omitted. Means and standard deviations for two independent experiments are shown, and mean differences between the controls are indicated.
